# IgE-Associated IGHV Genes from Venom and Peanut Allergic Individuals Lack Mutational Evidence of Antigen Selection

**DOI:** 10.1371/journal.pone.0089730

**Published:** 2014-02-25

**Authors:** Yan Wang, Katherine J. L. Jackson, Janet Davies, Zhiliang Chen, Bruno A. Gaeta, Janet Rimmer, William A. Sewell, Andrew M. Collins

**Affiliations:** 1 School of Biotechnology and Biomolecular Sciences, University of New South Wales, Sydney, Australia; 2 The Lung and Allergy Research Centre, School of Medicine, The University of Queensland, Woolloongabba, Australia; 3 School of Computer Science and Engineering, University of New South Wales, Sydney, Australia; 4 St. Vincent's Clinic, Darlinghurst, Australia; 5 Institute of Laboratory Medicine, St Vincent's Hospital, Darlinghurst, Australia and St Vincent's Clinical School, University of New South Wales, Darlinghurst, Australia; Cincinnati Children's Hospital Medical Center, University of Cincinnati College of Medicine, United States of America

## Abstract

Antigen selection of B cells within the germinal center reaction generally leads to the accumulation of replacement mutations in the complementarity-determining regions (CDRs) of immunoglobulin genes. Studies of mutations in IgE-associated VDJ gene sequences have cast doubt on the role of antigen selection in the evolution of the human IgE response, and it may be that selection for high affinity antibodies is a feature of some but not all allergic diseases.

The severity of IgE-mediated anaphylaxis is such that it could result from higher affinity IgE antibodies. We therefore investigated IGHV mutations in IgE-associated sequences derived from ten individuals with a history of anaphylactic reactions to bee or wasp venom or peanut allergens. IgG sequences, which more certainly experience antigen selection, served as a control dataset.

A total of 6025 unique IgE and 5396 unique IgG sequences were generated using high throughput 454 pyrosequencing. The proportion of replacement mutations seen in the CDRs of the IgG dataset was significantly higher than that of the IgE dataset, and the IgE sequences showed little evidence of antigen selection. To exclude the possibility that 454 errors had compromised analysis, rigorous filtering of the datasets led to datasets of 90 core IgE sequences and 411 IgG sequences. These sequences were present as both forward and reverse reads, and so were most unlikely to include sequencing errors. The filtered datasets confirmed that antigen selection plays a greater role in the evolution of IgG sequences than of IgE sequences derived from the study participants.

## Introduction

IgE class switching is generally considered a textbook demonstration of the Th2 response in action [1]. This class switching by a small number of B cells is accompanied by a parallel, more widespread switching to the IgG isotype, and these events are said to occur within the germinal centers of the lymph nodes, during the clonal expansion of antigen-selected B cells. This clonal expansion is accompanied by the accumulation of somatic point mutations within the variable regions of the immunoglobulin genes through a targeted mutation process [2], and the germinal center facilitates selection of mutated cells with improved antigen binding, leading to the production of higher affinity antibodies [3]. In recent years, a number of animal models have challenged this classical view of the IgE response. IgE production has been described in T cell deficient and MHC deficient mouse strains, with IgE class switching and some somatic mutation even occurring in animals that lack lymph nodes and Peyer's patches [4].

IgE class switching certainly occurs in murine lymph nodes, but Erazo and colleagues have highlighted the early departure of IgE-switched B cells from the GC, and their rapid differentiation into antibody-secreting plasma cells [5]. Such IgE-committed cells could either arise by direct class switching from IgM, or by sequential switching from recently switched IgG-committed cells. Others have described two pathways to IgE production in the mouse, with highly mutated high affinity IgE being generated through sequential class switching (IgM→IgG→IgE) and less mutated low affinity IgE being generated through direct class switching (IgM→IgE) [6].

These insights into IgE biology provide a new perspective from which to consider the unusual features of human IgE antibody gene sequences that we and others have reported. IgE-derived IGHV genes from non-allergic individuals have been shown to carry significantly fewer somatic point mutations in their IgE than in their IgG counterparts [7]. A surprising number of IgE sequences are relatively unmutated, and even sequences that totally lack somatic point mutations have been reported in allergic individuals [7], [8]. Studies of IgE gene sequences have also highlighted unexpected patterns of somatic point mutations. It has been argued that antigen selection should lead to an accumulation of replacement (R) mutations rather than silent (S) mutations within the complementarity determining regions (CDR) of rearranged immunoglobulin genes. This signature of selection has been absent in most [7], [9], [10], but not all [11] studies of IgE sequences from allergic individuals. Recently it was reported that selection is evident in IgE sequences from individuals with allergic asthma, but absent from sequences associated with atopic dermatitis [8], and it is therefore possible that sequences arising in different circumstances may be generated through the maturation of cells along alternative developmental pathways.

Previous studies of mutation patterns in IgE sequences have focused upon individuals suffering from allergic rhinitis, dermatitis and asthma. In contrast to these more usual allergic responses to ubiquitous environmental allergens, anaphylaxis can result from percutaneous exposure to ‘injectable’ allergens or from mucosal exposure to certain food allergens [12]. We hypothesized that the power of the anaphylactic response could be the result of high affinity IgE-producing B cells that emerge from the germinal center reaction. We therefore studied IgE sequences obtained from individuals with histories of anaphylactic reactions to bee or wasp venom or to peanut allergens.

The investigation of IgE gene sequences is challenging, for IgE-committed B cells are extremely rare, even in allergic individuals. In addition, a general lack of diversity in the IgE repertoire means that the generation of sufficient unique sequences for analysis can require the investigation of hundreds of clones, because of the dominance of certain replicate amplicons [8]. The characterization of IgE antibody genes has therefore been both expensive and time-consuming. High throughput sequencing (HTS) is a recent technical development that can efficiently generate thousands of unique reads, and long-read 454 HTS has transformed the study of the immunoglobulin repertoire [13]–[16]. However, 454 sequencing has not been used to investigate somatic point mutations, perhaps because it is assumed that the 454 error rate is unacceptably high for this purpose. We report here that HTS data is suitable for the analysis of the process of somatic point mutation, and although the specificities of the encoded antibodies are undeterminable, we demonstrate that IgE sequences from these individuals with venom and peanut allergies lack mutational evidence of antigen selection.

## Materials and Methods

### Ethics Statement

This study was approved by both the Royal North Shore Hospital and the UNSW Human Research Ethics Committees, and written consent was obtained from all participants in the study.

### Sample Collection and DNA template preparation

Blood samples were collected from ten adult donors with histories of anaphylactic reactions, though no donor was known to have experienced a very recent exposure to their sensitizing allergens. Donors included 4 patients with severe allergic sensitisation to bee or wasp venom and 6 patients with similarly severe sensitisation to peanut. Peripheral blood mononuclear cells (PBMC) were isolated by density gradient centrifugation using Ficoll-Paque PLUS (Amersham Biosciences). Total cellular RNA was then extracted using TRIzol™ Reagent, and cDNA synthesis was performed using Superscript®III Reverse Transcriptase (Invitrogen) and oligo(dT) primer.

### PCR and 454 sequencing

IgE sequences were amplified using semi-nested PCR and IgG sequences were amplified using conventional PCR. The primers used are shown in Table I. The forward primers were designed from FR1 sequences of the IGHV1, IGHV3 and IGHV4 families as these three families typically are present in over 90% of rearranged VDJ genes. The reverse primers for IgE PCR1 (ECH2R) and PCR2 (ECH1R) were designed from the CH2 and the CH1 regions of the IGHE gene, respectively. The reverse primer for the IgG PCR (GCHR) was designed from a conserved CH1 region of the four IgG constant region genes (IGHG1-4). GS FLX Titanium Primer A and Primer B sequences (Table I) were added to the 5′ end of the Forward and Reverse template-specific-sequence primers, respectively. Multiplex Identifiers sequences (MIDs) were also added between the GS FLX Titanium Primers and template-specific-sequence primers. All primers were synthesized by Integrated DNA Technologies (IDT).

**Table I:** Template-specific primer sequences for 454 sequencing.

Primer Name	Sequences (5′- 3′)	Description
VH1F	CAGRTSCAGCTGGTGCAGTCTGGG	IGHV1 forward primer
VH3F	ARGTGCAGCTGGTGGAGTCTGG	IGHV3 forward primer
VH4FA	AGSTGCAGCTGCAGGAGTCGG	IGHV4 forward primer 1
VH4FB	TACAGCAGTGGGGCGCAGGA	IGHV4 forward primer 2
ECH2R	GGACGACTGTAAGATCTTCACGG	IgE reverse primer for PCR1
ECH1R	GAATGTTTTTGCAGCAGCGGGT	IgE reverse primer for PCR2
GCHR	GGAAGTAGTCCTTGACCAGGCAG	IgG reverse primer
Primer A	CGTATCGCCTCCCTCGCGCCATCAG	GS FLX Titanium Primer A
Primer B	CTATGCGCCTTGCCAGCCCGCTCAG	GS FLX Titanium Primer B

Sequences were amplified using the FastStart High Fidelity PCR System (Roche). The IgE PCR1 reaction conditions were 95°C for 3 min, followed by 40 cycles of 95°C for 30 s, 60°C for 30 s and 72°C for 1 min, and then a final extension at 72°C for 5 min. In PCR2, reaction conditions were 95°C for 3 min, followed by 10 cycles of 95°C for 30 s, 64°C for 30 s and 72°C for 45 s, and then a final extension at 72°C for 5 min. The IgG PCR reaction conditions were 95°C for 3 min, followed by 30 cycles of 95°C for 30 s, 60°C for 30 s and 72°C for 35 s, and then a final extension at 72°C for 5 min.

PCR products were cleaned by gel extraction using QIAquick Gel Extraction Kits (QIAgen), and 454 sequencing was then performed at the Ramaciotti Centre for Gene Function Analysis, University of New South Wales on a Roche Genome Sequencer (GS) FLX using the GS FLX Titanium Sequencing kit.

### Bioinformatic analysis

The Smith-Waterman algorithm was used to identify duplicate sequences and to identify the isotype of each sequence. Where duplicate sequences were identified, if they varied in length, only the longest sequence was retained for analysis. Sequences were then partitioned with the iHMMune-align program [17], against a germline gene repertoire containing all unique IGHV, IGHD and IGHJ genes in the UNSWIg repertoire [18] (http://www.ihmmune.unsw.edu.au/unswig.php) and the IMGT repertoire [19]. The IGHV, IGHD and IGHJ gene names, and V sequence alignment, as well as nucleotide mismatches, insertions and deletions were recorded for each sequence.

Because of the use of FR1 region-specific primers, IGHV sequences were all truncated at the 5′ end. Any sequence that was missing more than 45 5′ nucleotides was removed from the dataset. Sequences with ambiguities, sequences containing insertions or deletions of nucleotides in the IGHV genes, as well as highly mutated sequences that contained more than 45 apparent mutations in the IGHV gene were also removed due to the likelihood that they included sequencing errors. Duplicate sequences were identified, and the number of replicates was noted before their removal from the dataset. This unfiltered dataset was designated Dataset A, and the sequences were deposited in the NCBI Sequence Read Archive (Project number: SRP033373).

All sequences that lacked bidirectional reads were removed from Dataset A and this filtered dataset was designated Dataset B. Clonally related sequences were then identified from their shared IGHV, IGHD and IGHJ genes, and shared CDR3 regions [20]. From each set of clonally-related sequences, the most abundant sequence was identified. Stringent filtering was then applied to yield a third dataset (Dataset C) in which all other clonally-related sequences beside the dominant sequence were removed. Differences in mutation levels between IgG and IgE sequences were analyzed by Student t test.

To investigate the role of antigen selection in the evolution of patterns of mutation within the IgE sequences, the proportion of replacement mutations within the CDR1 and CDR2 of each sequence was calculated. Broad definitions of CDR1 and CDR2 that incorporated the CDR regions of both Kabat [21] and IMGT [22] were used in the analysis, and comparison was made to a random model of mutations as previously described [23]. In the random model, the probability of a replacement mutation in the CDR was estimated to be 0.26. This was based upon analysis of mutations and mutational hotspots in a data set of non-productive sequences [23]. Using the binomial distribution, this probability was used to define 95% confidence limits for the proportion of the total mutations that would be replacement mutations in the CDR (R_CDR_), if mutations were not subject to antigen selection pressure. Proportions were calculated for varying numbers of total IGHV mutations (Mv), and the upper limit (97.5%) was used to distinguish sequences that showed evidence of antigen selection from sequences that lacked such evidence.

## Results

In order to study patterns of mutation in IgE heavy chain genes, IgE-derived VDJ rearrangements were generated from ten individuals, by 454 sequencing. So that comparisons could be made to sets of antigen-selected sequences, IgG-derived VDJ rearrangements were also generated from five of the ten individuals. The 53,688 amplicons included 31,248 IgE sequences, 16,980 IgG sequences and 5,460 sequences of uncertain isotype. After the removal of short sequences, sequences containing ambiguities, sequences containing insertions or deletions of nucleotides in the IGHV genes, duplicate sequences and sequences with greater than 45 mismatches to the most similar germline gene, 6025 IgE sequences and 5396 IgG sequences remained as Dataset A.

Sequences for which bidirectional reads had been obtained were then grouped as Dataset B, and this dataset included 577 IgE sequences and 482 IgG sequences. Clonally-related sequences were identified, and after removal of all but the most abundant sequence from each clone set, Dataset C contained 90 IgE sequences and 411 IgG sequences. The number of IgE sequences in each dataset that came from venom sensitive and peanut sensitive individuals are shown in Table II.

**Table II:** Numbers of IgE and IgG sequences in Datasets A, B and C.

	IgE (Venom, Peanut)^d^	IgG	Total
Total reads	31248	16980	53688
Dataset A^a^	6025 (2303, 3722)	5396	11421
Dataset B^b^	577 (262, 315)	482	1059
Dataset C^c^	90 (33, 57)	411	501

a All unique reads, after removal of duplicate sequences.

b All sequences in Dataset A that were seen as bidirectional reads.

c All sequences remaining after removal from Dataset B of all but the dominant sequences of clonally-related sets.

d Sequences derived from venom-allergic and peanut-allergic individuals.

Analysis of IGHV, IGHD and IGHJ genes in the VDJ rearrangements was performed using the iHMMune-align program. VDJ rearrangements were seen that utilized a very wide range of IGHV, IGHD and IGHJ germline genes. All IGHD and IGHJ genes were seen amongst the IgE and IgG sequences of each dataset, and all but the most rarely-rearranged IGHV genes of the IGHV1, IGHV3 and IGHV4 families were also seen in Dataset A. Sequences utilizing the IGHV5-51, IGHV5-a and IGHV7-4-1 genes were also present in each dataset. Biases in gene usage could only be considered in Dataset C, after the removal of clonally-related sequences, but these datasets were too small for meaningful analysis of gene usage.

In order to evaluate the quality of the sequences in the three datasets, out-of-frame (OOF) sequences and sequences that contained stop codons were identified. Although such sequences can be naturally generated, and small numbers of them can be expected in any dataset of VDJ rearrangements, their number will increase because of 454 sequencing errors. As shown in Table III, stringent filtering reduced the percentage of sequences that included stop codons from 6.5% in Dataset A, to 1.0% in Dataset B. There were no such sequences in Dataset C. Similarly, Dataset A included 13.6% OOFs, Dataset B had 3.4% OOFs, while Dataset C had only 1.0% OOFs (Table III).

**Table III:** Numbers of sequences of possibly low quality^a^ in unfiltered (A) and filtered (B and C) datasets.

	Dataset A	Dataset B	Dataset C
Sequences with Stop Codons	741 (6.5%)	11 (1.0%)	0 (0.0%)
Out of frame sequences (OOF)	1556 (13.6%)	36 (3.4%)	5 (1.0%)

a Sequences with stop codons and OOF sequences could arise naturally, but could be the result of sequencing errors.

### Mutation analysis

**Figure 1:**
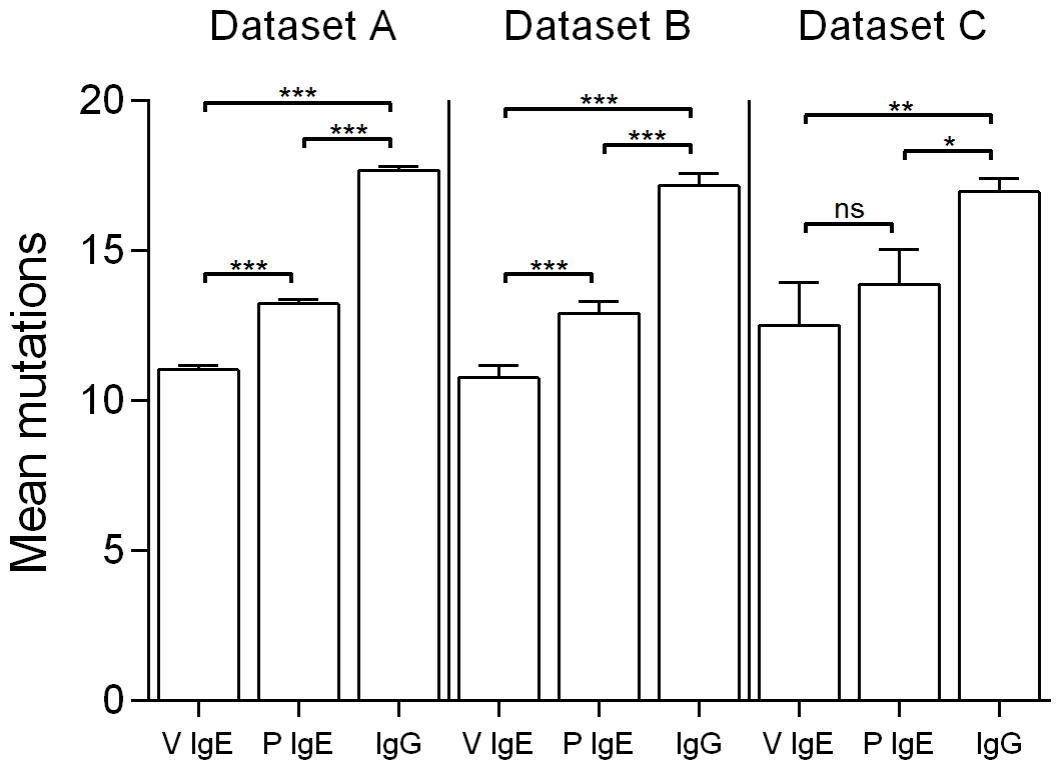
Mean numbers of mutations in IGHV genes derived from IgE-associated amplified from venom allergic (V IgE), peanut allergic donors (P IgE) and IgG for Dataset A, B and C. The exact specificities encoded by the IgE and IgG sequences are unknown.

The mean numbers of mutations in IgE and IgG sequences were calculated from the output of the iHMMune-align program. IgE-associated IGHV genes derived from venom-allergic and peanut-allergic individuals in Dataset A had mean mutation numbers of 11.0 (4.2%) and 13.2 (5.0%) respectively, while IgG-associated IGHV sequences had a mean 17.7 (6.7%) mutations (Figure 1). The difference in mutational levels between IgE sequences of the two patient groups was significant (p<0.01), but because of the possibility of distortions arising from clone sets of related sequences, the analysis was repeated using Datasets B and C. The differences between the IgE sequences of the two patient groups in Dataset C were not significant. The IgG sequences were significantly more mutated than both sets of IgE sequences (p<0.01 for Datasets B; p<0.05 for Dataset C).

**Figure 2:**
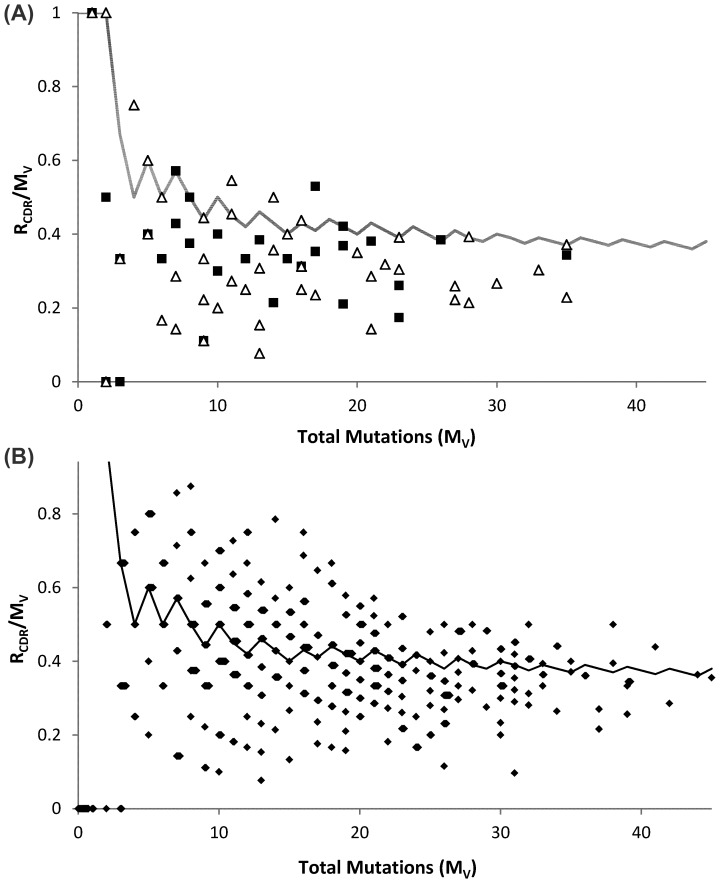
The proportion of total IGHV mutations (Mv) that are replacement mutations within the CDR1 and CDR2 regions (R_CDR_). The proportions of Mv that are R_CDR_ mutations are plotted against the total numbers of mutations in IgE sequences of unknown specificity, but derived from Venom (▪, n = 33) and Peanut (△, n = 57) allergic patients (A), and in IgG sequences (n = 411) (B). The solid lines show the 97.5% confidence limits for the R_CDR_/M_V_ ratio in a model of random mutation, where p(R_CDR_) equals 0.26. Data points have been adjusted to highlight clusters of overlaid values, and points above the line of the random model are considered to show evidence of antigen selection.

**Figure 3:**
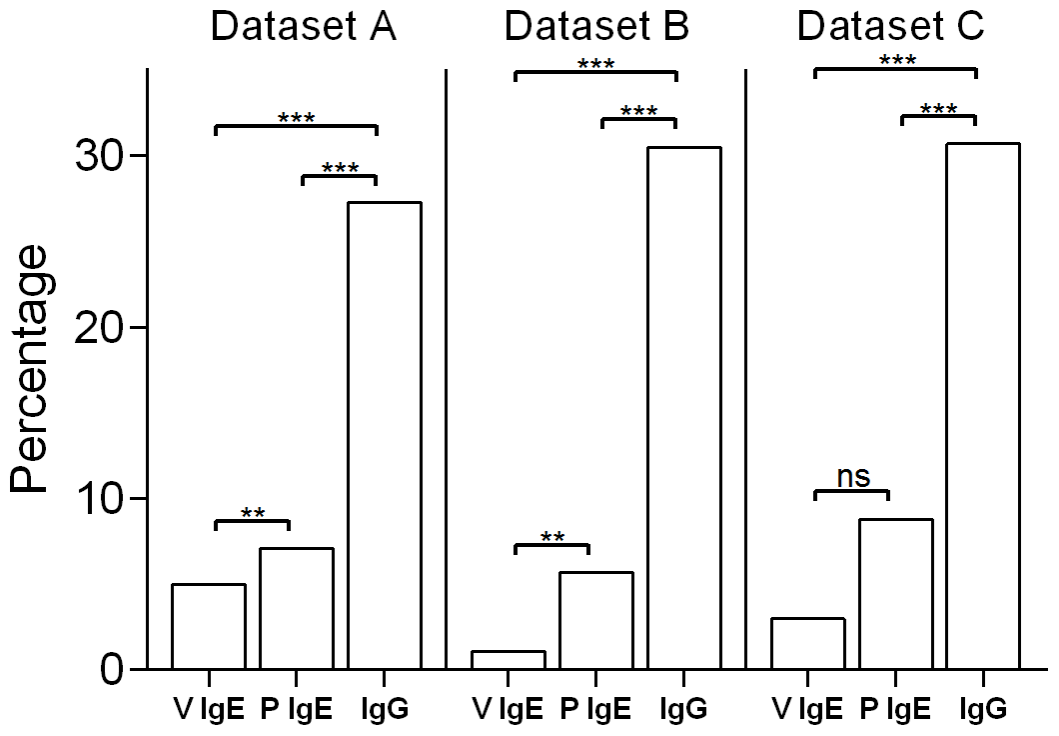
The percentages of sequences in Datasets A, B and C that showed evidence of antigen selection.

A comparison of the distribution of replacement mutations in sequences in Dataset C from venom and peanut allergic individuals showed there were no significant differences between patient groups for either IgE or IgG sequences. Analysis of the patterns of mutation of the combined IgE dataset showed that only 6.7% of IgE sequences (3.0% of sequences derived from bee venom allergic donors and 8.8% of sequences derived from peanut allergic donors) showed evidence of selection (Figures 2a and 3)). This was significantly fewer than the 30.7% of IgG sequences that showed evidence of antigen selection (chi square test: *p*<0.01) (Figure 2b and 3), ‘Selected’ sequences show a higher proportion of replacement mutations in the CDR than expected, based upon the random model of mutation, and appear ‘above the line’ in Figure 2.

## Discussion

Almost fifteen years after it was first suggested that IgE sequences carried distinct patterns of somatic point mutations, the role of antigen selection in the evolution of the human IgE response remains a contentious issue [24], [25]. It was recently argued that the use of high throughput sequencing would be necessary if the issue is to be resolved [25]. It was argued that only high throughput sequencing would deliver the large data sets that are required for meaningful mutation analysis [25]. In addition, it was argued that HTS makes it possible to deduce the germline repertoire of each individual studied. This would reduce errors in the identification of mutations that otherwise result from inaccuracies in the identification of the IGHV gene within each VDJ gene rearrangement [25].

The danger of misidentification of an individual's IGHV genes, within VDJ rearrangements, is illustrated by an early study that reported a small number of IgE sequences amplified from the peripheral blood of two peanut allergic individuals [26]. In a reanalysis of the seventeen sequences, we found that only two sequences had the same number of replacement mutations in the CDR regions as was originally reported (data not shown). The errors in the original report likely reflect the state of knowledge of the germline gene repertoire at that time. The original study relied upon the early V Base dataset [27]. In the intervening years, there has been a substantial expansion [18], [28], [29] as well as revision [30] of the IGHV germline gene repertoire.

In this study, we have used 454 pyrosequencing to generate both IgE and IgG sequences from the peripheral blood of venom and peanut allergic individuals. Although 454 sequencing was not used to genotype the IGH locus in the subject participants, the number of sequences was sufficient to allow us to review the datasets for the presence of previously unreported polymorphisms. None were identified, and we can be confident that mutation numbers were accurately determined. The inclusion of IgG sequences provided an additional control, and an essential point of comparison. We assumed that antigen selection guided the evolution of these sequences. This was borne out by the analysis, and challenges the recent claim that current tests lack the sensitivity to detect antigen selection even in IgG sequences [25].

Although evidence of antigen selection was only seen in 31% of IgG sequences, the contrast with IgE sequences was striking and highly significant. In the most carefully filtered datasets, only 3% of sequences derived from venom allergic donors and 9% of sequences derived from peanut allergic donors showed evidence of antigen selection. The specificity of the IgE sequences in this study could not be determined, and it is possible that they encode IgE antibodies directed against other unknown allergens. It was also not possible to investigate the impact of either the IgG or IgE mutations on the antigen affinity of the antibodies. Nevertheless, this study adds to the growing body of evidence that suggests that VDJ genes associated with the IgE response are the products of polyclonal activation of B cells, or of some other unconventional process outside the germinal center reaction.

Previous reports of a lack of evidence of antigen selection and an apparent bias in the IGHV gene repertoire in IgE sequences led to the suggestion that allergic IgE may arise from superantigen-like polyclonal activation of B cells by some allergens [31]. It has also been suggested that low allergen concentrations, or the absence of danger signals during allergen exposure, could lead to the formation of ‘immature’ germinal centres which could favor the development of IgE antibodies, and that the unusual patterns of R and S mutations seen in allergic IgE sequences could result from different selection processes in such immature germinal centers [32]. Others have argued that unusual distributions of mutations in allergic IgE sequences could reflect persistent stimulation at mucosal sites, where class switching to IgE and somatic point mutation are now known to occur [10], [33]. In light of recent observations in mouse models, the possibility must now be accepted that class switching to IgE in humans may also be followed by the rapid exit of switched cells from the germinal center reaction. It may be that this separate developmental pathway leads to the distinct patterns of silent and replacement mutations seen in IgE sequences.

The existence of a separate developmental pathway for at least some IgE-switched cells raises the possibility that there may be additional complexity to the developmental pathways of cells of other isotypes within the germinal center reaction. From an analysis of somatic point mutations in VDJ rearrangements associated with different IgG isotypes, we recently proposed a Temporal Model of IgE and IgG subclass function [34]. Analysis of the numbers and patterns of somatic point mutations is a novel new approach to the experimental investigation of B cell development and B cell function, and investigation of the Temporal Model will require detailed investigations of somatic mutations in large numbers of antibodies produced by both healthy and diseased individuals. It is therefore important that the present study has shown that high throughput sequencing can provide an efficient means of conducting such investigations, despite its relatively high error rate.
